# Post-treatment late and long-term effects in bone sarcoma: A scoping review

**DOI:** 10.1016/j.jbo.2025.100671

**Published:** 2025-03-21

**Authors:** Kaainat Khan, Kathleen Kane, Zoe Davison, Darrell Green

**Affiliations:** a Biomedical Research Centre, Norwich Medical School, University of East Anglia, Norwich Research Park, Norwich, United Kingdom; b Bone Cancer Research Trust, Leeds, United Kingdom

**Keywords:** Bone sarcoma, Cancer, Late effects, Chemotherapy, Survivorship

## Abstract

Despite the fact that chemotherapy for bone sarcomas (e.g. Ewing sarcoma, osteosarcoma) has well-reported toxicities and that surgical intervention is frequently life altering, follow-up care to monitor for late and long-term effects beyond that of oncological surveillance in former patients is variable. Anecdotal evidence suggests that inconsistent follow-up means some former bone sarcoma patients are left to cope with post-treatment late and long-term effects with limited support. Here, we performed a scoping review to provide a more empirical identification of the knowledge gaps and to provide an overview of the peer reviewed academic literature reporting the late and long-term effects of treatment for bone sarcoma. JBI Scoping Review Network guidelines for charting, analysis and data extraction were followed. Literature searches were conducted in Medline (Ovid), Cochrane CENTRAL, EMBASE (Ovid), CINAHL, PsycINFO, Proquest and Web of Science (Clarivate Analytics) from March 2024 to September 2024. Paper titles and abstracts were screened by two independent reviewers followed by full text analysis by the lead researcher. Seventy-four peer reviewed articles were included in the analysis. Most studies were of a retrospective study design, some up to 20 years of follow-up and included chemotherapy, surgery and sometimes radiotherapy as the treatment modality. Our analysis identified secondary malignancies, cardio- and nephrotoxicity, lower bone mineral density and microarchitectural deterioration, cancer related fatigue and motor neuropathies as the major physical late and long-term effects requiring dedicated follow-up. In some cases, follow-up may need to span decades, especially given the increasing population of former patients. Our results form the evidence-based foundations for future work that might include late and long-term effect follow-up service mapping exercises and expanded clinical recommendations.

## Highlights

• Bone sarcomas are better considered a systemic disease with radiologically undetectable micrometastases already present at diagnosis.
• Chemotherapy was added to the clinical protocol in the 1970s. Actuarial 10-year survival rates improved significantly from 10% to 40%.
• Despite the success of chemotherapy, the agents used have known toxicities that can cause long-term health effects in former patients.
• Our review of the peer reviewed literature shows secondary malignancies, skeletal complications and organ failure require dedicated check-up.
• Continued follow-up of former bone sarcoma patients, beyond that of oncological surveillance, is essential to improve overall patient care.

## 1 Introduction

Ewing sarcoma and osteosarcoma are malignant bone tumours mostly diagnosed in children, adolescents and young adults [1], [2], [3], [4]. As almost all patients with bone sarcomas developed metastases in the short term with local therapy alone, chemotherapy was added to the clinical protocol in the 1970s [5], [6], [7], [8]. Local radiotherapy was included for some Ewing sarcoma cases [9]. Combining local and systemic therapy led to significant improvements in survival outcomes. Actuarial 10-year survival rates increased from 10 % to 40 % and even higher in localised disease cases [9], [10]. Despite the success of chemotherapy, it has well-reported toxicities that can cause long-term health effects in former patients (a preferred term used by some members of the bone sarcoma community instead of ‘survivor’). Empirical evaluation of outcomes following treatment including functioning and quality of life have become critical to investigate because former patients have voiced their frustrations with cancer aftercare. This problem is ever expanding given the increasing population of former patients and requires an evidence-based approach to lay some groundwork for more dedicated assessment in the future. Here, we performed a scoping review of the peer reviewed academic literature reporting the late and long-term effects of the chemotherapy protocol for bone sarcomas (vincristine, doxorubicin and cyclophosphamide alternating with ifosfamide and etoposide (VDC/IE) for Ewing sarcoma; methotrexate, cisplatin and doxorubicin (MAP) for osteosarcoma), radiotherapy and wide margin surgery, revealing treatment implications and the subsequent effects in former patients’ lives after interventional care has been delivered.

## 2 Methods

### 2.1 Data sources

JBI Scoping Review Network guidelines for charting, analysis and data extraction were followed [11], [12]. As per JBI’s scoping review manual, *a priori* protocol was curated before the review was initiated. The PCC mnemonic for population, concept and context was applied as a question development framework to expand the outcome of interest. For this study, the outcomes were (i.) primary bone cancer that fits as a population qualifying criteria, (ii.) late/long-term effects including physical, psychological or social and (iii.) supportive needs or aftercare. Points ii. and iii. were the core concepts examined by the scoping review that articulated the scope and breadth of inquiry. Cultural factors including geographical location, racial or sex-based interests were not applied because the supportive needs for every former patient are required to be addressed. For this reason, no literature were ruled out or excluded from the searches because all key findings were essential to map out the available evidence and bolster future research in this area.

### 2.2 Inclusion criteria

A comprehensive multifield search was applied using the following databases: EMBASE, Medline (OVID), Web of Science (Clarivate Analytics), Cochrane CENTRAL, CINAHL APA, PsycINFO and Proquest. Since no exclusion criteria were defined prior to the literature review, the approach taken when using the databases was to search for available literature through ‘Boolean logic’ to combine search terms related to the area of this investigation. Different variations of these phrases were combined or excluded to produce relevant literature (Table 1). The studies eligible for inclusion included synonyms of “*primary bone cancer*” combined with equivalences of “*late effects*” or “*survivorship*” and “*follow-up*” (‘‘*needs’’*) or “*aftercare*”. The search was performed without restrictions for date or methodologies. Articles were deemed eligible if they identified risk factors of treatment type, treatment year or whether tumour related type/histology influenced the occurrence of late effects. Patient demographics, social and lifestyle factors were important to observe and were included. Quantitative studies such as population-based studies and qualitative reports studying the physical and psychosocial late effects in patients with bone sarcoma were included along with guidelines and systematic reviews. As per the methodology of scoping reviews, the quality or risk of bias were not appraised [12].

**Table 1:** The strategy for literature searches used Boolean logic to define relationships between terms in the search. The Boolean search operators and/or were used to create a broad search and encompass all available literature. The search terms were combined to result in a search that contained all terms and led to the most relevant literature.

#	Searches
	Primary bone cancer OR bone sarcoma* OR Ewing sarcoma OR osteosarcoma OR chondrosarcoma OR chordoma OR adamantinoma etc.
AND	Late effect* OR long term OR longer term OR survivorship
AND	Need* OR support* OR after* OR after-care OR follow* OR follow-up

### 2.3 Study selection

**Fig. 1:**
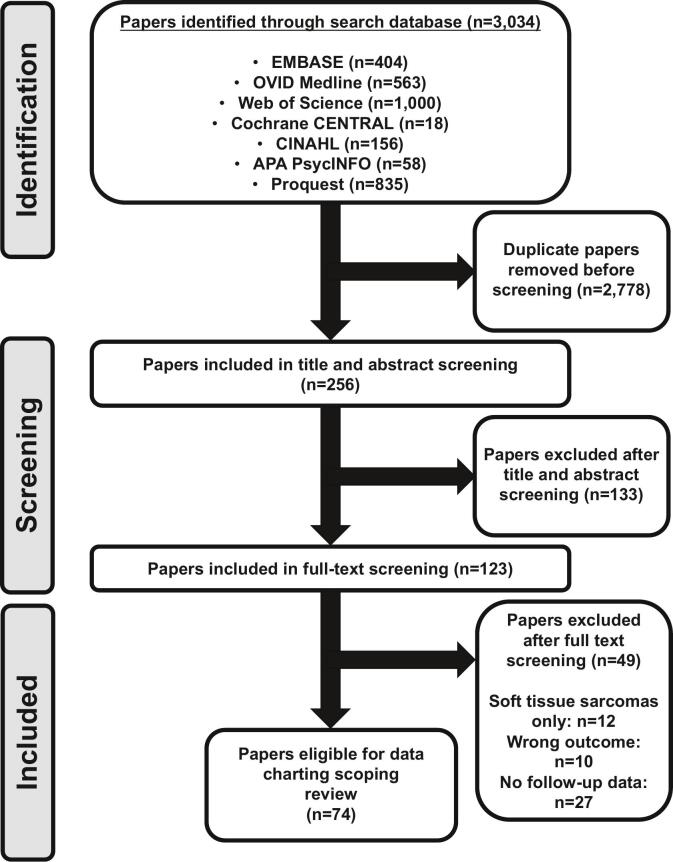
Search strategy used. Seventy-four papers were eligible for data extraction and descriptive analysis.

The initial selection process began with screening of titles and abstracts with the support of two independent reviewers to minimise selection bias in the first instance. Full text retrievals based on potentially relevant evidence were selected for further review if they met the criteria defined *a priori*. Title, abstract and full text screening led to eligible record collection (Fig. 1). The study selection process of included articles was completed through narrative analysis and a brief synopsis of the appraised evidence plus descriptive statistics rather than assessment through *meta*-analysis due to data heterogeneity [13]. Following duplicate exclusion, a full text screening was performed on 74 independent studies (Table 2).

**Table 2:** Article characteristics used in the scoping review.

Characteristics of articles included in the scoping review (n = 74)			
	Late Effects	Physical Late Effects	Psychosocial Late Effects
**Databases**	n of papers	n of papers	n of papers
EMBASE	12	4	2
MEDLINE	3	13	3
Web of Science	1	10	
Cochrane CENTRAL		2	
CINAHL	3	5	2
PsycINFO		1	6
Proquest	1	3	3
**Total papers**	20	38	16
**Patients**	13,223	89,000	1312
**Study design**	n of papers	n of papers	n of papers
Retrospective	7	30	4
Cross-sectional	12	5	12
Prospective	1	3	
**Sample size**	n of papers	n of papers	n of papers
<50	8	16	9
50–100	1	9	5
>100	10	11	2
>1000		1	
>10,000	1	1	
**Bone sarcoma types**	% of papers	% of papers	% of papers
Osteosarcoma	70 %	52 %	75 %
Ewing sarcoma	65 %	65 %	81 %
Chondrosarcoma	30 %	26 %	56 %
Chordoma	30 %	13 %	6 %
Adamantinoma		2 %	
Multiple bone sarcomas	10 %	7 %	6 %
**Age at diagnosis**	n of papers	n of papers	n of papers
<10 years	1	1	
11–20 years	6	12	4
21–30 years		1	
31–40 years			
41–50 years		1	
>60 years	1		
Unknown	9	18	8
Broad range	3	5	4
**Treatment**	n of papers	n of papers	n of papers
Chemotherapy	1	8	
Radiotherapy	1	4	
Surgery	2	4	2
Chemotherapy + Radiotherapy	4	4	
Chemotherapy + Surgery	2	2	
Chemotherapy + Radiotherapy + Surgery	10	15	14
Radiotherapy + Surgery		1	
**Follow-up duration**	n of papers	n of papers	n of papers
<2 years	1	5	3
2–5 years	2	3	3
>5 years	17	30	10
**Region**			
Argentina		1	
Austria		1	1
Australia	1		3
Belgium		1	
Canada	2	1	
China		1	
Egypt			1
Europe	1	5	1
France		1	
Germany		1	2
Italy	2	8	
India		1	
Japan		1	
Netherlands	3	1	
Norway	4	1	3
Russia			1
Spain		2	
Sweden		1	
Switzerland			1
Turkey	1	1	
Thailand		1	
United Kingdom	2	2	
United States	4	7	3

## 3 Results

### 3.1 Data charting process

The database searches produced 3,034 articles following the search strategy (Fig. 1; Table 1). After removing 2,778 duplicates, 256 studies were screened and 123 were selected for full text review based on their relevance to the research question: what the empirical evidence for late and long-term effects is requiring follow-up independent to oncological surveillance. Further reading of the full texts narrowed down the selected papers to 74. Some studies were excluded due to wrong study type, outcome or no follow-up data recorded. The 74 papers were eligible for data extraction and descriptive analysis. Most studies were retrospective and focussed on physical late effects (n = 30) (Table 3). The most widely used treatment modality was a combination of chemotherapy, surgery and radiotherapy (n = 39) with a follow-up of > 5 years (Table 2). Across all of the studies reviewed, 78 % considered the physical late effects and 48 % considered psychosocial late effects (Table 4). Breakdown of the age groups investigated shows almost one-third of studies focussed exclusively on former patients aged 11–20 y at their bone sarcoma diagnosis. Forty-seven percent of studies did not specify age at diagnosis.

**Table 3:** Physical late effect characteristics.

Physical Late Effects (n = 58)								
Category	Number of papers	No. of participants in study	Year of study	Study design	Diagnosis	Treatment modality	Follow-up time	Physical late effects
*Secondary malignancies*	19	7,262	1992–2023		Osteosarcoma		9 months − > 20 years	Subsequent malignant neoplasms
					11			(n = 17),
				Cross-sectional	Ewing sarcoma	Chemotherapy		Breast metastases
				4	10	14		(n = 2),
				Prospective	Chondrosarcoma	Radiotherapy		Bone metastases
				2	3	15		(n = 7),
				Retrospective	Chordoma	Surgery		Pulmonary metastases
				13	3	11		(n = 8),
					Multiple bone sarcomas			
					1			
*Cardiotoxicity*	14	83,946	2004–2023		Osteosarcoma		< 2 years − 30 years	Cardiac toxicity
					10			(n = 12),
				Cross-sectional	Ewing sarcoma	Chemotherapy		Cardiac dysfunction
				2	9	14		(n = 1),
				Prospective	Chondrosarcoma	Radiotherapy		Left ventricular dysfunction
					2	9		(n = 2),
				Retrospective	Chordoma	Surgery		Cardiomyopathy
				12	2	7		(n = 2),
					Multiple bone sarcomas			Heart disease
					1			(n = 2),
*Nephrotoxicity*	9	1,012	1998–2020		Osteosarcoma		< 2 years − > 20 years	Proximal tubular dysfunction
					6			(n = 1),
				Cross-sectional	Ewing sarcoma	Chemotherapy		Tubulopathy
				2	4	8		(n = 2)
				Prospective	Chondrosarcoma	Radiotherapy		Renal toxicity
				1	1	5		(n = 2)
				Retrospective	Chordoma	Surgery		Glomerular damage
				6	1	3		(n = 1)
					Multiple bone sarcomas			Reduced renal function
					1			(n = 5)
*Orthopaedic complications*	8	322	2012–2024		Osteosarcoma		5 years − 20 years	Low bone mineral density
					5			(n = 4),
				Cross-sectional	Ewing sarcoma	Chemotherapy		Microarchitectural deterioration
				2	6	7		(n = 1)
				Prospective	Chondrosarcoma	Radiotherapy		Bone reabsorption
					1	4		(n = 1)
				Retrospective	Chordoma	Surgery		Limb length defects
				6	1	2		(n = 3)
					Multiple bone sarcomas			Osteopenia / Osteoporosis
								(n = 4)
*Fatigue*	9	747	1999–2023		Osteosarcoma		3 months − 20 years	
					5			Effect of radiotherapy
				Cross-sectional	Ewing sarcoma	Chemotherapy		(n = 4),
				7	6	7		Effect of chemotherapy
				Prospective	Chondrosarcoma	Radiotherapy		(n = 2)
				1	5	7		Dizziness
				Retrospective	Chordoma	Surgery		(n = 1)
				1	5	7		Chronic fatigue
					Multiple bone sarcomas			(n = 7)
					1			
*Hearing loss, memory, vertigo*	7	1,191	2009–2023		Osteosarcoma		2 years − 20 years	Impaired hearing
					5			(n = 4),
				Cross-sectional	Ewing sarcoma	Chemotherapy		Memory loss
				4	4	6		(n = 1)
				Prospective	Chondrosarcoma	Radiotherapy		Vertigo
					2	5		(n = 2)
				Retrospective	Chordoma	Surgery		Ototoxicity
				3	1	2		(n = 2)
					Multiple bone sarcomas			Cognitive impairment
								(n = 2)
*Fertility issues*	8	1,290	1992–2021		Osteosarcoma		< 2 years − > 20 years	
					4			Infertility
				Cross-sectional	Ewing sarcoma	Chemotherapy		(n = 8)
				4	5	7		Premature menopause
				Prospective	Chondrosarcoma	Radiotherapy		(n = 1)
					1	6		Sterile patients
				Retrospective	Chordoma	Surgery		(n = 1)
				4		4		Gonadotoxicity
					Multiple bone sarcomas			(n = 1)
					1

**Table 4:** Psychosocial late effect characteristics.

Psychosocial Late Effects (n = 36)								
Category	No. of papers	No. of participants in study	Year of study	Study design	Diagnoses	Treatment modality	Follow-up time	Physical late effects
*Psychology*	14	558	1995–2021		Osteosarcoma		< 2 years − 24 years	Existential considerations
					11			(n = 4),
				Cross-sectional	Ewing sarcoma	Chemotherapy		Body image concerns
				9	10	10		(n = 2),
				Prospective	Chondrosarcoma	Radiotherapy		Chronic psychological distress
					5	9		(n = 2),
				Retrospective	Chordoma	Surgery		Impairments of neurocognition
				5	1	11		(n = 1),
					Multiple bone sarcomas			Post-traumatic stress disorder
								(n = 2),
*Social integration and finance*	8	782	1995–2021		Osteosarcoma			
					7		3 years – 16 years	Employment / Education
				Cross-sectional	Ewing sarcoma	Chemotherapy		(n = 3),
				3	6	5		Return to work
				Prospective	Chondrosarcoma	Radiotherapy		(n = 2)
					1	4		Finances
				Retrospective	Chordoma	Surgery		(n = 2)
				5		4		Social media concerns
					Multiple bone sarcomas			(n = 1)
					1			
*Quality of life*	9	1101	1995–2023		Osteosarcoma			
					6		4 months – 40 years	
				Cross-sectional	Ewing sarcoma	Chemotherapy		Poor HRQOL
				4	6	6		(n = 1)
				Prospective	Chondrosarcoma	Radiotherapy		Loss of vitality
				1	5	7		(n = 1)
				Retrospective	Chordoma	Surgery		Chronic pain
				4	1	7		(n = 2)
					Multiple bone sarcomas			
					1			

### 3.2 Secondary malignancies

Found to be of particular concern amongst former patients through their subjective responses on a questionnaire was the potential higher risk for developing a subsequent/second primary malignancy (e.g. bone, breast, leukaemia) following their treatment for bone sarcoma [14]. Clinically reported evidence of secondary malignancies or a subsequent malignant neoplasm (SMN) appeared in 19 studies assessing the late consequences of bone sarcoma treatment; examined between 1992 and 2023, with 70.6 % of studies investigating individuals ≤18 y at age of diagnosis, whilst 29 % included adult survivors [14], [15], [16], [17], [18], [19], [20], [21], [22], [23], [24], [25], [26], [27], [28], [29], [30], [31], [32]. This important finding endorses that former bone sarcoma patients are at a higher risk of SMN development.

Assessment of long-term outcomes for former osteosarcoma patients found that the treatment factor attributing most to SMN development was a higher cumulative exposure to the platinum-based agent cisplatin [16]. For former Ewing sarcoma patients, radiation therapy was the largest contributing factor even at lower doses (<50 Gy) and might occur very late (e.g. 20-years after original treatment) [30]. Radiation associated SMN cases increased with longer follow-up schedules [22]. Investigation of cause specific mortality for 664 former bone sarcoma patients showed that they are four times more likely to develop an SMN than is expected (compared to non-treated individuals) during a 24-year follow-up; however, after this time, the risk reduces to expected cancer incidence levels [14].

Alkylating agents used for Ewing sarcoma treatment such as cyclophosphamide and etoposide were found to lead to the development of acute myeloid leukaemia (AML) from three to ten years post-treatment [21]. Incidence increased parallelly with the number of chemotherapy agents used. A longitudinal follow-up study of former Ewing sarcoma patients, now in adulthood, characterised AML as the most common SMN along with secondary osteosarcomas, breast and thyroid cancer [21].

### 3.3 Cardiotoxicity

Cardiotoxic effects arising from systemic treatments were reported in 14 studies highlighting this as another important late effect [16], [17], [21], [28], [32], [33], [34], [35], [36], [37], [38], [39], [40], [41]. Another consequence to cisplatin use, along with its association to SMNs, is the cardiac toxicity that high cumulative exposure exerts [16]. Anthracyclines, also integral components to the osteosarcoma treatment protocol, are known to have cardiotoxic side effects [34].

Patients who undergo amputation surgeries are prone to loss of physical movement and therefore may experience barriers to exercise, which in the longer term also contributes as a risk factor for cardiovascular events [37]. In a study reporting the experience of the Italian Sarcoma Group, of the 883 patients with osteosarcoma, 18 patients (2 %) experienced cardiomyopathy and half (9) died from congestive heart failure. Among the 9 survivors, 4 had to undergo a heart transplant [17].

In a large cohort study of sarcoma patients, patients diagnosed at an older age were more prone to develop heart failure due to the co-morbidities of ageing combined with toxicities from chemotherapy [41]. Older patients were less likely to have long-term surveillance [36].

### 3.4 Nephrotoxicity

Ifosamide is used for the treatment of osteosarcoma (front-line in France, second line in the UK) and for the front-line treatment (international) of Ewing sarcoma. Nephrotoxicity was reported as a significant side effect in 7 studies investigating those aged <18 y at diagnosis (43 % of studies) [32], [34], [38], [39], [41], [42], [43]. Damage to the proximal tubules can result in a loss of phosphate, bicarbonate, glucose, amino acids and low weight proteins, which can lead to renal Fanconi syndrome in serious cases [42]. Older patients were more likely to experience renal tubulopathy and increased urinary excretion [34]. Cisplatin, ifosfamide and high-dose methotrexate were all found to be linked to renal toxicity with cisplatin potentiating ifosamide-induced damage [34]. The EURO-B.O.S.S. study reported that nephrotoxicity incidence was higher in those >40 y than for younger patients leading to new guidance recommending a modified MAP regime for patients >40 y (ifosfamide instead of high-dose methotrexate) [44].

### 3.5 Skeletal complications

Bone sarcoma treatment in children can impair the attainment of peak bone mass that predisposes to the onset of low bone mineral density (BMD) [45]. Microarchitectural deterioration can persist into adulthood and increase fracture risk [45], [46]. Adolescent patients who have not yet undergone a pubertal growth spurt or epiphyseal plate closure at diagnosis can experience skeletal late effects such as osteopenia, osteoporosis and fractures [46]. Lower BMD was found in 58 % of patients in a study investigating 43 patients with Ewing sarcoma and osteosarcoma who underwent chemotherapy [47]. Vitamin D deficiency was also found as a late effect of chemotherapy [47]. Supplementation should be considered post-treatment [48]. Patients aged < 10 y at diagnosis may develop short stature [49].

A review of 4 studies found that immobilisation and decreased physical activity (because of treatment intensity) causes bone resorption leading to decreased bone mass and a higher fracture risk [45]. Disease located in the lower extremity showed local osteopenia conditions [50]. This observation thematically links to the late effect of osteoporosis that was found in a study investigating 24 former patients diagnosed at an adult age [34]. Musculoskeletal symptoms including abnormal gait and negative joint function were observed in one-third of patients (8/24) who underwent prosthetic joint replacement or limb amputation [34]. In 207 former patients of Ewing sarcoma and osteosarcoma (now in adulthood), more than one-third had radiographic evidence of peripheral sensory neuropathy and increased motor neuropathies [35].

### 3.6 Cancer related fatigue

A side effect found to persist during particle radiotherapy treatment was tiredness and fatigue [51], [52]. The creation of a simple form by the Norwegian Clinical Sarcoma Research group facilitated useful communication with patients on long-term consequences of their treatments. From this study, which included 54 patients with bone sarcomas, fatigue was the most frequent late effect raised (39 % of patients) and was noted to appear more often in patient responses when compared to patients with soft tissue sarcomas [53]. Quality of life for former patients who received an allograft fusion and/or endoprosthesis (limb sparing surgery) was poorer than those who received amputation or rotationplasty in terms of fatigue in the long term [54]. Fatigue was experienced by 28 % of patients who underwent surgical treatment for malignant and benign bone tumours, suggesting surgical intervention as a potential causative factor [55]. Fatigue was also reported in a study of 102 patients who received chemotherapy for Ewing sarcoma or osteosarcoma [56].

### 3.7 Impaired hearing, memory loss and vertigo

Impaired hearing and memory loss were reported as a late effect of radiation toxicity [51]. In a study investigating 34 former bone sarcoma patients, diffusion magnetic resonance imaging of the cerebral white matter combined with the Wechsler Adult Intelligence Scale (WAIS) intelligence test, age at diagnosis and assessment and time since diagnosis showed that chemotherapy was associated with reduced nerve fiber density in the cingulum and corpus callosum plus with attention deficits, memory loss and reduced processing speeds [57]. Hearing loss has been linked with high cisplatin doses [16]. Cisplatin increased the prevalence of vertigo, reported in 14 % of 733 former osteosarcoma patients [16]. A study by the Scandinavian Sarcoma Group found 33 % of former patients experienced ototoxicity after treatment [43]. As evaluated by audiograms, hearing loss was detected in 40 % of patients with osteosarcoma after a cumulative cisplatin dose of 600 mg/m^2^ [28].

### 3.8 Fertility issues

In males, abnormal sperm concentrations were frequently reported [35]. Fertility was impaired in 47 out of 54 (87 %) male patients with osteosarcoma who underwent sperm analysis post-treatment [32]. Of 207 female patients with osteosarcoma, 6 had impaired fertility [32]. A qualitative interview study exploring the late consequences for 8 patients with osteosarcoma found chemotherapy was the leading cause of fertility complications [58]. Patients who received cyclophosphamide as part of their treatment reported fewer pregnancies and impaired fertility [35].

### 3.9 Physical limitations

Although limb salvage surgery has functional and cosmetic advantages over amputation surgery, there can be psychosocial and functional consequences for patients with bone sarcomas. In 18 former osteosarcoma patients with impaired physical function and reduced mobility, half articulated concerns about the visible differences associated with functional impairment [59]. Patients often felt the need to hide their bodily changes, to feel as normal as possible, as well as to feel healthy and physically (i.e. sexually) attractive [59]. Participants in a comparative qualitative study of limb salvage and amputation surgery stated that regardless of whether they underwent amputation or limb salvage surgery, those with more functional lower limbs had a better quality of life than those with less functional lower limbs [60]. This finding could be related to diminished locoregional functioning, which can limit occupational opportunities and in turn socioeconomic health for patients [33]. Amongst 664 former patients, problems with activities such as walking (22 %), bathing or dressing (21 %) were almost four times higher than those for the general population (5 %), which further emphasises the physical health implications following bone sarcoma treatment [61].

### 3.10 Psychological impact

Just under half of the examined studies briefly considered the psychosocial and more holistic patient concerns including psychological impact, social integration, financial difficulties and quality of life. Lowered self-esteem was a major psychological implication reported by 18 patients as a long-term consequence of completing treatment for bone sarcoma in their hip/pelvis region or lower extremities [58]. Patients reported cosmetic or visual consequences including limps, scars or skin discoloration being an important aspect of discontent [58], [62]. Body image concerns were rated significantly worse for patients who underwent late amputation surgery and amputation after limb salvage [60].

In exploring the different trajectories that former patients navigate during follow-up three to ten years after diagnosis, there were three different rehabilitation phrases identified amongst the 18 participants: “*back to normal*”, “*a new normal*“ and “*still struggling*” [63]. The majority (15/18) of study participants considered their lives and their bodies to be considerably different (“*a new normal*”). It was deemed amongst the former patients to be strenuous and time-consuming to adopt a new identity as a person with disabilities so “*new normal*” was the avenue to cope [63]. The participants agreed that they would have benefitted from dedicated and tailored psychological follow-up. Two patients reported being “*stuck*” in a situation where the late and long-term effects of their bone sarcoma treatment impacted their everyday lives to the extent that they no longer considered their lives to be meaningful [63]. In another study, around 30 % of all patients with sarcoma experienced clinically significant levels of distress with many meeting the criteria for a post-traumatic stress diagnosis [64].

### 3.11 Social integration and financial difficulties

The demands of cancer treatment often conflict with adolescent and young adult developmental necessities such as increased independence and peer interaction. For an adolescent or young adult with bone sarcoma these conflicts are magnified due to high symptomatic burden and late effects of the invasive treatments. One qualitative study showed that former young adult patients often experience loneliness and have difficulty integrating with friendship groups [65]. In their study of 39 former patients, Nurdan et al. found that osteosarcoma treatment effects led to significantly less likelihood to obtain/attain educational qualifications, marital status, employment and parenthood [28]. In another study, however, 80 % of patients revealed only minor psychosocial problems, being able to adapt to their new living conditions [66], though there were differences compared to the control group with regards to marital status, independent living and parenthood [66], [67].

Returning to work is a major step for former patients after treatment. Some former patients used work as an approach to return to their prior known structures of everyday life [68]. Return to work in some cases was challenging since verbal comments from colleagues plus changes in performance became a reminder of their status prior to diagnosis [68].

Patients with bone sarcomas can also experience financial difficulties. Not working, not receiving benefits and the ongoing costs of medication and parking in hospitals were reported issues in an interview study of health professionals working with sarcoma (n = 21), patients diagnosed with sarcoma (n = 22) and carers of patients diagnosed with sarcoma (n = 17) [69].

### 3.12 Quality of life

Given the overall reduced physical functioning of former bone tumour patients, there is a compromise to health related quality of life [67]. In the long-term follow up of 18 former patients, 3 highlighted a loss of their main hobby, an active or athletic activity and struggled more with the change of being devoid of physical activity, contrasting to the life they once enjoyed. These concessions led to patients suffering from a lack of motivation, fatigue, reduced cognitive function and mental health challenges, which ultimately impacted their quality of life [63]. Fatigue amongst 170 patients was associated with psychological variables including reduced optimism [55]. Significant rates of chronic psychological distress were found in psychiatric evaluations of now-adult former Ewing sarcoma patients exhibiting major depression, alcohol abuse and post-traumatic stress disorder [33], [35].

For 23 former patients who underwent limb salvage surgery, there was a general reluctancy to share feelings of body image issues and mobility difficulties with their oncologist due to concerns of appearing vain or unappreciative [70]. This and other studies emphasise that global function and reintegration into “*normal*“ living plays a role in quality of life [16]. Not speaking about their frustrations often led to patients adopting social isolation practices and avoidant coping strategies, which further impacted their mental health and functional quality of life [70].

## 4 Discussion

The term ‘cancer survivor’ is broad and variously defined, encompassing people living with cancer, in remission or those with no evidence of disease. It is predicted that by 2030 almost one million people in the UK (1 in 70 people) will be living with moderate to severe physical and psychological effects following cancer treatment [71]. The survivorship trajectory starts at diagnosis and continues through life. Some people do not identify with the term ‘survivor’ because the treatment effects become a new health burden in themselves [72]; however, ‘survivorship care’ is intended to improve health, wellness and quality of life. This care type focuses on the wide and lifelong impact of cancer and its treatment. Care addresses physical and mental health, health behaviours, personal and professional identity and finances. Many cancer treatment effects such as nausea and vomiting quickly resolve after treatment ends. Some effects do not resolve, or new effects start after treatment. ‘Long-term effects’ start during treatment and persist when treatment has concluded; for example, cognitive problems and fatigue. ‘Late effects’ occur long after treatment has ended; for example, second cancers [73], [74].

Models for survivorship care to address these late and long-term effects vary across cancer types, healthcare contexts and continents. In 2008, the National Cancer Survivorship Initiative (NCSI) was launched in the UK. The aim of this partnership, which is a collaboration between Macmillan, NHS Improvement and the Department of Health and Social Care, is to better understand the cancer survivorship experience and to develop models capable of addressing the needs of those living with and beyond cancer [75]. The goal of this collaboration was to bring about a cultural shift in the UK healthcare system’s approach to survivorship care. This objective led to significant progress in the development of follow-up pathways with headway made for breast, prostate and colorectal cancers in particular, though ongoing resource and government commitment are needed to achieve lasting and more widespread implementation [76]. Internationally, the North American National Comprehensive Cancer Network (NCCN) provides detailed consensus-based guidelines for follow-up monitoring of late and long-term effects. A working group of the European PanCareSurFup Consortium (PanCare Childhood and Adolescent Cancer Survivor Care and Follow-up Studies) have developed evidence-based recommendations for the organisation of long-term follow up care for child and adolescent patients [77]. In the context of bone sarcoma, NHS England’s sarcoma service specifications and UK and international clinical guidelines include recommendations for late and long-term effects monitoring [9], [78]. Yet, qualitative evidence gathered across small-scale studies with the UK bone sarcoma community suggests that varying access to such inconsistent follow-up means some former patients are left to cope with the late and long-term effects with limited support [79], [80].

We have performed a scoping review to provide an overview of the peer reviewed and published literature describing the late and long-term effects of treatment for bone sarcomas and to identify the knowledge gaps in this area. The work was intended to provide an empirical foundation for future work that might include late and long-term effects mapping exercises and research to inform dedicated clinical recommendations on follow-up following treatment for bone sarcoma, which in some cases may need to span decades. A number of peer reviewed articles (n = 74) were analysed for this evidence-based investigation. There was no restriction to geographical location because cancer treatment late effects are universal for all individuals. Grey literature (defined as information produced by non-commercial entities such as charities, pressure groups, government agencies, preprints, conference proceedings and doctoral theses, which are not indexed in major academic databases) was not considered so that the potential risk for limited interpretability of results was reduced.

Beyond oncological surveillance and recurrence of the primary disease, the most compromising physical late and long-term effects were secondary malignancies, cardio- and nephrotoxicity and skeletal complications. These morbidities require urgent intervention if detected, highlighting the importance for regular and dedicated follow-up independent to relapse/recurrence surveillance. Heart disease caused by non-bacterial thrombotic endocarditis, chemo- and radiotherapy exposure have become the largest underlying cause of death in former patients with cancer [37].

A proposition for the detection of cardiotoxic late effects is to evaluate whether patients present with symptoms of heart failure, an arrythmia or general cardiac abnormalities during late effects follow-up. Reduced systolic function can be detected with routine monitoring during and post-treatment. Diagnostic echocardiography images, cardiovascular magnetic resonance imaging or nuclear quantification of left ventricular ejection fraction can also elucidate systolic dysfunction and cardiotoxicity [81].

There is no uniform diagnostic screening for nephrotoxicity but the Acute Kidney Injury Network (AKIN) [82] proposed measuring abrupt changes in serum creatinine or urine as new diagnostic criterion for determining the incidence and severity of nephrotoxicity [83]. It should be noted that this specific screening still warrants confirmation from a large prospective trial [83].

Differentiation and identification between osteoporosis and low bone mineral density can be assessed through radiomorphometric indices and cone beam computed tomography scans [84]. Fatigue, memory loss and vertigo effects can be investigated through clinical examinations and history taking during follow-up consultations. Regular otoscopy and audiometry tests should be conducted to monitor for impaired hearing late effects [85]. Fertility can be assessed through ovarian and testicular assessments, MRI and pelvic/testicular ultrasound imaging [86], [87].

Literature on the physical late effects (reported in 78 % of included studies) were more prevalent than psychosocial late effects (reported in 48 % of included studies) but it is important to note from the evidence that the physical implications including reduced mobility or chronic fatigue suggest a correlation to negative psychology and social functioning. The aetiology of cancer related fatigue is most commonly encountered in patients who underwent radiotherapy [52], which is significant because there may be causality between fatigue and depression [88]. Disease progression over time can lead to a delayed onset of post-traumatic stress [89] with additional psychological implications as well as physical manifestations such as chronic musculoskeletal pain, hyperlipidaemia, hypertension and cardiovascular disease [89]. There is an interplay and sequential impact from both physical and psychosocial late effects that clinicians should be aware of in order to assess this, routinely, so its disabling impacts on patient functioning can be mitigated.

The importance of providing post-treatment psychological support appears to be less recognised in the literature. One study of a small group (n = 23) found that former patients are typically left to deal with their mental wellbeing in isolation [70]. Since bone sarcomas are mostly diagnosed in the 2nd and 3rd decades, fear and uncertainty about the future can continue to be a significant concern for former patients, particularly those in the adolescent and young adult (AYA) age groups. There is no current consensus on how to address the schedule for psychological surveillance in AYAs; however, developing proactive screening tools to differentiate those patients who require assistance from those at risk of psychological distress are imperative for limiting late psychological complications [90]. Ensuring care is age appropriate is vital since premature confrontation with mortality, fertility issues and body concerns can lead to an unprepared realisation of the disruption resultant from treatment complications later in life [90]. Age-appropriate follow-up is essential to communicate these apprehensions effectively and although heterogeneity in patient populations can make it difficult to assess quality of life in AYA patients, approaches for routine systemic screening are underway [91].

A main limitation of the available literature was a lack of investigation into late effects in middle-aged patients (30–50 y) and in older patients (>60 y). Literature pertaining to Ewing sarcoma and osteosarcoma (n = 45) were more extensive when compared to chondrosarcoma, chordoma and adamantinoma late effects (n = 37). Throughout the research there is an underassessment of older patients who have been treated for bone sarcoma, leading to a lack of evidence relating to experiences of late and long-term effects in this age group specifically. More primary research in these areas are required.

The British Sarcoma Group's UK guidelines for the management and follow up of bone sarcomas (primary bone cancers) state that: *“It is important to evaluate the long-term toxicity of chemotherapy and radiotherapy as well as immediate chemotherapy-related complications. Monitoring for late effects should be undertaken, depending on the treatment and in conjunction with available late effect services”* [9]. The European Society for Medical Oncology guidelines for bone sarcomas also state: “*Long-term toxic effects of chemotherapy, surgery and radiotherapy should be evaluated and monitoring for late effects should be continued for > 10 years after treatment, depending on the protocol used*” [78]. Given the breadth of late and long-term effects identified in this scoping review, more specific detail relating to clinical management of the individual toxicity effects may be warranted in future versions of clinical guidelines.

## 5 Conclusion

This scoping review has established an evidence base that supports the need for dedicated long-term follow up and late effects monitoring, which goes beyond oncological surveillance (e.g. relapse/recurrence) following treatment for bone sarcoma. Identification of a diverse range of physical toxicities impacting cardiac and renal functioning as well as the risk of secondary malignancy and the potential for skeletal complications, fertility issues and deficits to hearing and cognitive functioning justify the need for wide-ranging screening delivered within a multidisciplinary model of survivorship care. The significant psychosocial implications, which are less represented across the available literature, emphasise the need for a holistic approach. The scoping review findings demonstrate how late effects can occur at any point during a former patients’ lifetime and therefore, ongoing follow-up of cancer survivors is essential to define specific groups at higher risk of complications, identify unrecognised long-term adverse effects and improve overall patient care. The work should be considered a critical first step in gathering the evidence needed to guide future prospective studies to further assess the key late and long-term effects drawn from the literature and to inform possible future improvements to long-term follow up care.

## CRediT authorship contribution statement

**Kaainat Khan:** Writing – original draft, Visualization, Validation, Resources, Methodology, Investigation, Formal analysis, Data curation. **Kathleen Kane:** Writing – review & editing, Validation, Supervision, Methodology, Investigation, Formal analysis, Data curation. **Zoe Davison:** Writing – review & editing, Validation, Supervision, Methodology, Investigation, Formal analysis. **Darrell Green:** Writing – original draft, Visualization, Validation, Supervision, Resources, Project administration, Methodology, Investigation, Formal analysis, Data curation, Conceptualization.

## Funding

This study did not receive any specific grant from funding agencies in the public, commercial or not-for-profit sectors.

## Declaration of competing interest

The authors declare that they have no known competing financial interests or personal relationships that could have appeared to influence the work reported in this paper.

## References

[1] Green D., van Ewijk R., Tirtei E., Andreou D., Baecklund F., Baumhoer D., Bielack S.S., Botchu R., Boye K., Brennan B., Capra M., Cottone L., Dirksen U., Fagioli F., Fernandez N., Flanagan A.M., Gambarotti M., Gaspar N., Gelderblom H., Gerrand C., Gomez-Mascard A., Hardes J., Hecker-Nolting S., Kabickova E., Kager L., Kanerva J., Kester L.A., Kuijjer M.L., Laurence V., Lervat C., Marchais A., Marec-Berard P., Mendes C., Merks J.H.M., Ory B., Palmerini E., Pantziarka P., Papakonstantinou E., Piperno-Neumann S., Raciborska A., Roundhill E.A., Rutkauskaite V., Safwat A., Scotlandi K., Staals E.L., Strauss S.J., Surdez D., Sys G.M.L., Tabone M.D., Toulmonde M., Valverde C., van de Sande M.A.J., Woertler K., Campbell-Hewson Q., McCabe M.G., Nathrath M. (2024). Biological sample collection to advance research and treatment: a Fight Osteosarcoma Through European Research (FOSTER) and Euro Ewing Consortium (EEC) statement. Clin. Cancer Res..

[2] Green D., Singh A., Tippett V.L., Tattersall L., Shah K.M., Siachisumo C., Ward N.J., Thomas P., Carter S., Jeys L., Sumathi V., McNamara I., Elliott D.J., Gartland A., Dalmay T., Fraser W.D. (2023). YBX1-interacting small RNAs and RUNX2 can be blocked in primary bone cancer using CADD522. J. Bone Oncol..

[3] Green D., Eyre H., Singh A., Taylor J.T., Chu J., Jeys L., Sumathi V., Coonar A., Rassl D., Babur M., Forster D., Alzabin S., Ponthan F., McMahon A., Bigger B., Reekie T., Kassiou M., Williams K., Dalmay T., Fraser W.D., Finegan K.G. (2020). Targeting the MAPK7/MMP9 axis for metastasis in primary bone cancer. Oncogene.

[4] Llaneza-Lago S., Fraser W.D., Green D. (2024). Bayesian unsupervised clustering identifies clinically relevant osteosarcoma subtypes. Brief. Bioinform..

[5] Frei E., Jaffe N., Watts H. (1978). Adjuvant chemotherapy of osteogenic sarcoma: progress and perspectives4. JNCI: Journal of the National Cancer Institute.

[6] Rosen G., Tan C., Sanmaneechai A., Beattie E.J., Marcove R., Murphy M.L. (1975). The rationale for multiple drug chemotherapy in the treatment of osteogenic sarcoma. Cancer.

[7] Cores E.P., Holland J.F., Wang J.J., Sinks L.F. (1972). Doxorubicin in disseminated osteosarcoma. J. Am. Med. Assoc..

[8] Jaffe N., Frei E., Traggis D., Bishop Y. (1974). Adjuvant methotrexate and citrovorum-factor treatment of osteogenic sarcoma. N. Engl. J. Med..

[9] Gerrand C., Amary F., Anwar H.A., Brennan B., Dileo P., Kalkat M.S., McCabe M.G., McCullough A.L., Parry M.C., Patel A., Seddon B.M., Sherriff J.M., Tirabosco R., Strauss S.J. (2024). UK guidelines for the management of bone sarcomas. Br. J. Cancer.

[10] Bielack S.S., Kempf-Bielack B., Delling G., Exner G.U., Flege S., Helmke K., Kotz R., Salzer-Kuntschik M., Werner M., Winkelmann W., Zoubek A., Jürgens H., Winkler K. (2002). Prognostic factors in high-grade osteosarcoma of the extremities or trunk: an analysis of 1,702 patients treated on neoadjuvant cooperative osteosarcoma study group protocols. J. Clin. Oncol..

[11] Tricco A.C., Lillie E., Zarin W., O'Brien K.K., Colquhoun H., Levac D., Moher D., Peters M.D.J., Horsley T., Weeks L., Hempel S., Akl E.A., Chang C., McGowan J., Stewart L., Hartling L., Aldcroft A., Wilson M.G., Garritty C., Lewin S., Godfrey C.M., Macdonald M.T., Langlois E.V., Soares-Weiser K., Moriarty J., Clifford T., Tunçalp Ö., Straus S.E. (2018). PRISMA extension for scoping reviews (PRISMA-ScR): checklist and explanation. Ann. Intern. Med..

[12] Peters M.D.J., Marnie C., Tricco A.C., Pollock D., Munn Z., Alexander L., McInerney P., Godfrey C.M., Khalil H. (2021). Updated methodological guidance for the conduct of scoping reviews. JBI Evid. Implement..

[13] Campbell M., Katikireddi S.V., Sowden A., McKenzie J.E., Thomson H. (2018). Improving conduct and reporting of narrative synthesis of quantitative data (ICONS-Quant): protocol for a mixed methods study to develop a reporting guideline. BMJ Open.

[14] Fidler M.M., Frobisher C., Guha J., Wong K., Kelly J., Winter D.L., Sugden E., Duncan R., Whelan J., Reulen R.C., Hawkins M.M. (2015). Long-term adverse outcomes in survivors of childhood bone sarcoma: the British Childhood Cancer Survivor Study. Br. J. Cancer.

[15] Nicholson H.S., Mulvihill J.J., Byrne J. (1992). Late effects of therapy in adult survivors of osteosarcoma and Ewing's sarcoma. Med. Pediatr. Oncol..

[16] Nagarajan R., Kamruzzaman A., Ness K.K., Marchese V.G., Sklar C., Mertens A., Yasui Y., Robison L.L., Marina N. (2011). Twenty years of follow-up of survivors of childhood osteosarcoma: a report from the Childhood Cancer Survivor Study. Cancer.

[17] Longhi A., Ferrari S., Tamburini A., Luksch R., Fagioli F., Bacci G., Ferrari C. (2012). Late effects of chemotherapy and radiotherapy in osteosarcoma and Ewing sarcoma patients: the Italian Sarcoma Group Experience (1983-2006). Cancer.

[18] Kaiser I., Kauertz K., Zöllner S.K., Hartmann W., Langer T., Jürgens H., Ranft A., Dirksen U. (2022). Secondary malignancies after ewing sarcoma-epidemiological and clinical analysis of an international trial registry. Cancers.

[19] Goedhart L.M., Ho V.K.Y., Ploegmakers J.J.W., van der Geest I.C.M., van de Sande M.A.J., Bramer J.A., Stevens M., Jutte P.C. (2023). Bone sarcoma follow-up; a nationwide analysis of oncological events after initial treatment. J Bone Oncol.

[20] Goedhart L.M., Leithner A., Ploegmakers J.J.W., Jutte P.C. (2020). Follow-up in bone sarcoma care: a cross-sectional European study. Sarcoma.

[21] Marina N.M., Liu Q., Donaldson S.S., Sklar C.A., Armstrong G.T., Oeffinger K.C., Leisenring W.M., Ginsberg J.P., Henderson T.O., Neglia J.P., Stovall M.A., Yasui Y., Randall R.L., Geller D.S., Robison L.L., Ness K.K. (2017). Longitudinal follow-up of adult survivors of Ewing sarcoma: A report from the Childhood Cancer Survivor Study. Cancer.

[22] Goldsby R., Burke C., Nagarajan R., Zhou T., Chen Z., Marina N., Friedman D., Neglia J., Chuba P., Bhatia S. (2008). Second solid malignancies among children, adolescents, and young adults diagnosed with malignant bone tumors after 1976: follow-up of a Children's Oncology Group cohort. Cancer.

[23] Gonzalez C.D., Randall R.L., Wright J., Spraker-Perlman H., Ying J., Sweeney C., Smith K.R., Kirchhoff A.C. (2017). Long-term survivors of childhood bone and soft tissue sarcomas are at risk of hospitalization. Pediatr. Blood Cancer.

[24] Cool P., Grimer R., Rees R. (2005). Surveillance in patients with sarcoma of the extremities. Eur. J. Surg. Oncol..

[25] Cotterill S.J., Ahrens S., Paulussen M., Jürgens H.F., Voûte P.A., Gadner H., Craft A.W. (2000). Prognostic factors in Ewing's tumor of bone: analysis of 975 patients from the European Intergroup Cooperative Ewing's Sarcoma Study Group. J. Clin. Oncol..

[26] Paulino A.C. (2004). Late effects of radiotherapy for pediatric extremity sarcomas. Int. J. Radiat. Oncol. Biol. Phys..

[27] York J.E., Kaczaraj A., Abi-Said D., Fuller G.N., Skibber J.M., Janjan N.A., Gokaslan Z.L. (1999). Sacral chordoma: 40-year experience at a major cancer center. Neurosurgery.

[28] Nurdan T., Hatice Mine C., Emel U., Handan D., Gulsah T., Incesoy Ozdemir S., Omer K., Yasin Y., Yusuf Y., Basarir K., Gulsan Y. (2021). Late effects of osteosarcoma and its treatment in pediatric patients: a single-center experience. J. B.U.ON. Off. J. Balkan Union Oncol..

[29] Sallabanda M., Vera J.A., Pérez J.M., Matute R., Montero M., de Pablo A., Cerrón F., Valero M., Castro J., Mazal A., Miralbell R. (2023). Five-fraction proton therapy for the treatment of skull base chordomas and chondrosarcomas: early results of a prospective series and description of a clinical trial. Cancers.

[30] Longhi A., Barbieri E., Fabbri N., Macchiagodena M., Favale L., Lippo C., Salducca N., Bacci G. (2003). Radiation-induced osteosarcoma arising 20 years after the treatment of Ewing's sarcoma. Tumori.

[31] Shanmugam S., Govindasamy G., Hussain S.A., Fells S.P.S. (2019). Pediatric bone sarcomas: outcome of multimodality treatment in a single institution in South India over a decade Indian. J. Med. Paediatr. Oncol..

[32] Longhi A., Pasini E., Bertoni F., Pignotti E., Ferrari C., Bacci G. (2004). Twenty-year follow-up of osteosarcoma of the extremity treated with adjuvant chemotherapy. J. Chemother. (Florence, Italy).

[33] Mansky P., Arai A., Stratton P., Bernstein D., Long L., Reynolds J., Chen D., Steinberg S.M., Lavende N., Hoffman K., Nathan P.C., Parks R., Augustine E., Chaudhry U., Derdak J., Wiener L., Gerber L., Mackall C. (2007). Treatment late effects in long-term survivors of pediatric sarcoma. Pediatr. Blood Cancer.

[34] van de Luijtgaarden A.C., Kapusta L., Bellersen L., Bokkerink J.P., Kaal S.E., Versleijen-Jonkers Y.M., Schreuder H.W., van der Graaf W.T. (2014). High prevalence of late adverse events in malignant bone tumour survivors diagnosed at adult age. Neth. J. Med..

[35] Bishop M.W., Ness K.K., Li C., Liu W., Srivastava D.K., Chemaitilly W., Krull K.R., Green D.M., Pappo A.S., Robison L.L., Hudson M.M., Mulrooney D.A. (2020). cumulative burden of chronic health conditions in adult survivors of osteosarcoma and ewing sarcoma: A report from the St. Jude Lifetime Cohort Study. Cancer Epidemiol. Biomarkers Prevent..

[36] Heemelaar J.C., Speetjens F.M., Al Jaff A.A.M., Evenhuis R.E., Polomski E.A.S., Mertens B.J.A., Jukema J.W., Gelderblom H., van de Sande M.A.J., Antoni M.L. (2023). Impact of Age at Diagnosis on Cardiotoxicity in High-Grade Osteosarcoma and Ewing Sarcoma Patients. JACC. Cardiooncol..

[37] Chen B., Zhao X., Li X., Liu J., Tang J. (2022). Fatal heart disease in patients with bone and soft tissue sarcoma. Front. Cardiovasc. Med..

[38] Zhang J., Huang Y., Sun Y., He A., Zhou Y., Hu H., Yao Y., Shen Z. (2019). Impact of chemotherapy cycles and intervals on outcomes of nonspinal Ewing sarcoma in adults: a real-world experience. BMC Cancer.

[39] Armenian S.H., Hudson M.M., Mulder R.L., Chen M.H., Constine L.S., Dwyer M., Nathan P.C., Tissing W.J., Shankar S., Sieswerda E., Skinner R., Steinberger J., van Dalen E.C., van der Pal H., Wallace W.H., Levitt G., Kremer L.C. (2015). Recommendations for cardiomyopathy surveillance for survivors of childhood cancer: a report from the International Late Effects of Childhood Cancer Guideline Harmonization Group. Lancet Oncol..

[40] Volkova M., Russell R. (2011). Anthracycline cardiotoxicity: prevalence, pathogenesis and treatment. Curr. Cardiol. Rev..

[41] Ferraresi V., Vari S., Rossi B., Maggi G., Giannarelli D., Persichetti A., Petrongari M.G., Cercato M.C., Annovazzi A., Anelli V., Pescarmona E., Baldi J., Zoccali C., Pellegrini D., Cognetti F., Biagini R. (2020). The real-life journey of elderly patients in soft tissue and bone sarcomas: a retrospective analysis from a sarcoma referral center. J. Clin. Med..

[42] Stöhr W., Paulides M., Bielack S., Jürgens H., Treuner J., Rossi R., Langer T., Beck J.D. (2007). Ifosfamide-induced nephrotoxicity in 593 sarcoma patients: a report from the Late Effects Surveillance System. Pediatr. Blood Cancer.

[43] Aksnes L.H., Bauer H.C., Dahl A.A., Fosså S.D., Hjorth L., Jebsen N., Lernedal H., Hall K.S. (2009). Health status at long-term follow-up in patients treated for extremity localized Ewing Sarcoma or osteosarcoma: a Scandinavian sarcoma group study. Pediatr. Blood Cancer.

[44] Ferrari S., Bielack S.S., Smeland S., Longhi A., Egerer G., Hall K.S., Donati D., Kevric M., Brosjö O., Comandone A., Werner M., Monge O., Palmerini E., Berdel W.E., Bjerkehagen B., Paioli A., Lorenzen S., Eriksson M., Gambarotti M., Tunn P.U., Jebsen N.L., Cesari M., Kalle T., Ferraresi V., Schwarz R., Bertulli R., Kasparek A.K., Grignani G., Krasniqi F., Sorg B., Hecker-Nolting S., Picci P., Reichardt P. (2018). EURO-B.O.S.S.: A European study on chemotherapy in bone-sarcoma patients aged over 40: outcome in primary high-grade osteosarcoma. Tumori.

[45] Marcucci G., Beltrami G., Tamburini A., Body J.J., Confavreux C.B., Hadji P., Holzer G., Kendler D., Napoli N., Pierroz D.D., Rizzoli R., Brandi M.L. (2019). Bone health in childhood cancer: review of the literature and recommendations for the management of bone health in childhood cancer survivors. Ann. Oncol..

[46] Holzer G., Hobusch G., Hansen S., Fischer L., Patsch J.M. (2021). Is there an association between bone microarchitecture and fracture in patients who were treated for high-grade osteosarcoma? A controlled study at long-term follow-up using high-resolution peripheral quantitative CT. Clin. Orthop. Relat. Res..

[47] Pirker-Frühauf U.M., Friesenbichler J., Urban E.C., Obermayer-Pietsch B., Leithner A. (2012). Osteoporosis in children and young adults: a late effect after chemotherapy for bone sarcoma. Clin. Orthop. Relat. Res..

[48] van Atteveld J.E., Verhagen I.E., van den Heuvel-Eibrink M.M., van Santen H.M., van der Sluis I.M., Di Iorgi N., Simmons J.H., Ward L.M., Neggers S. (2021). Vitamin D supplementation for children with cancer: A systematic review and consensus recommendations. Cancer Med..

[49] Hoshi M., Oebisu N., Iwai T., Ban Y., Nakamura H. (2022). Does systemic chemotherapy influence skeletal growth of young osteosarcoma patients as a treatment-related late adverse effect?. Curr. Oncol. (Toronto Ont.).

[50] Müller C., Winter C.C., Rosenbaum D., Boos J., Gosheger G., Hardes J., Vieth V. (2010). Early decrements in bone density after completion of neoadjuvant chemotherapy in pediatric bone sarcoma patients. BMC Musculoskelet. Disord..

[51] Fauske L., Jebsen N.L., Bondevik H., Bruland S. (2023). Exploring the patient perspective of bone sarcoma survivors who have undergone particle radiotherapy abroad. Anticancer Res..

[52] Srivastava A., Vischioni B., Fiore M.R., Vitolo V., Fossati P., Iannalfi A., Tuan J.K., Orecchia R. (2013). Quality of life in patients with chordomas/chondrosarcomas during treatment with proton beam therapy. J. Radiat. Res..

[53] Hompland I., Fauske L., Lorem G.F., Bruland S. (2020). Use of a simple form to facilitate communication on long-term consequences of treatment in sarcoma survivors. Clin. Sarcoma Res..

[54] Barrera M., Teall T., Barr R., Silva M., Greenberg M. (2012). Health related quality of life in adolescent and young adult survivors of lower extremity bone tumors. Pediatr. Blood Cancer.

[55] Servaes P., Verhagen S., Schreuder H.W., Veth R.P., Bleijenberg G. (2003). Fatigue after treatment for malignant and benign bone and soft tissue tumors. J. Pain Symptom Manage..

[56] Sirikul W., Buawangpong N., Pruksakorn D., Charoentum C., Teeyakasem P., Koonrungsesomboon N. (2023). The survival outcomes, prognostic factors and adverse events following systemic chemotherapy treatment in bone sarcomas: a retrospective observational study from the experience of the cancer referral center in Northern Thailand. Cancers.

[57] Sleurs C., Lemiere J., Christiaens D., Billiet T., Peeters R., Sunaert S., Uyttebroeck A., Deprez S. (2018). Advanced MR diffusion imaging and chemotherapy-related changes in cerebral white matter microstructure of survivors of childhood bone and soft tissue sarcoma?. Hum. Brain Mapp..

[58] Fauske L., Bruland O.S., Grov E.K., Bondevik H. (2015). Cured of primary bone cancer, but at what cost: a qualitative study of functional impairment and lost opportunities. Sarcoma.

[59] Fauske L., Lorem G., Grov E.K., Bondevik H. (2016). Changes in the body image of bone sarcoma survivors following surgical treatment–A qualitative study. J. Surg. Oncol..

[60] Robert R.S., Ottaviani G., Huh W.W., Palla S., Jaffe N. (2010). Psychosocial and functional outcomes in long-term survivors of osteosarcoma: a comparison of limb-salvage surgery and amputation. Pediatr. Blood Cancer.

[61] Reulen R.C., Winter D.L., Lancashire E.R., Zeegers M.P., Jenney M.E., Walters S.J., Jenkinson C., Hawkins M.M. (2007). Health-status of adult survivors of childhood cancer: a large-scale population-based study from the British Childhood Cancer Survivor Study, International journal of cancer. J. Internat. Du Cancer.

[62] Bekkering W.P., Vliet Vlieland T.P., Koopman H.M., Schaap G.R., Schreuder H.W., Beishuizen A., Tissing W.J., Hoogerbrugge P.M., Anninga J.K., Taminiau A.H. (2010,). Quality of life in young patients after bone tumor surgery around the knee joint and comparison with healthy controls. Pediat. Blood Cancer.

[63] Fauske L., Bondevik H., Ahlberg K., Bjørndal A. (2019). Identifying bone sarcoma survivors facing psychosocial challenges. A study of trajectories following treatment. Eur. J. Cancer Care.

[64] Storey L., Fern L.A., Martins A., Wells M., Bennister L., Gerrand C., Onasanya M., Whelan J.S., Windsor R., Woodford J., Taylor R.M. (2019). A critical review of the impact of sarcoma on psychosocial wellbeing. Sarcoma.

[65] Donovan E., Martin S.R., Seidman L.C., Zeltzer L.K., Cousineau T.M., Payne L.A., Knoll M., Weiman M., Federman N.C. (2021). The role of social media in providing support from friends for adolescent and young adult (AYA) patients and survivors of sarcoma: perspectives of AYA, parents, and providers. J. Adol. Young Adult Oncol..

[66] Felder-Puig R., Formann A.K., Mildner A., Bretschneider W., Bucher B., Windhager R., Zoubek A., Puig S., Topf R. (1998). Quality of life and psychosocial adjustment of young patients after treatment of bone cancer. Cancer.

[67] Eiser C. (2009). Assessment of health-related quality of life after bone cancer in young people: easier said than done. Eur. J. Cancer (Oxford, England: 1990).

[68] Zambrano S.C., Kollár A., Bernhard J. (2020). Experiences of return to work after treatment for extremital soft tissue or bone sarcoma: Between distraction and leaving the disease behind. Psychooncology.

[69] Weaver R., O'Connor M., Sobhi S., Carey Smith R., Halkett G. (2020). The unmet needs of patients with sarcoma. Psychooncology.

[70] Taylor M.F., Pooley J.A. (2017). Sarcoma survivors' perspectives on their body image and functional quality of life post-resection/limb salvage surgery. Eur. J. Cancer Care.

[71] Marzorati C., Riva S., Pravettoni G. (2017). Who is a cancer survivor? A systematic review of published definitions. J. Cancer Educat..

[72] Khan N.F., Harrison S., Rose P.W., Ward A., Evans J. (2012). Interpretation and acceptance of the term 'cancer survivor': a United Kingdom-based qualitative study. Eur. J. Cancer Care.

[73] Harrington J., White J. (2017). The late medical effects of cancer treatments: a growing challenge for all medical professionals. Clin. Med. (Lond.).

[74] Stein K.D., Syrjala K.L., Andrykowski M.A. (2008). Physical and psychological long-term and late effects of cancer. Cancer.

[75] Richards M., Corner J., Maher J. (2011). The National Cancer Survivorship Initiative: new and emerging evidence on the ongoing needs of cancer survivors. British Journal of Cancer 105 Suppl.

[76] Maher J., Petchey L., Greenfield D., Levitt G., Fraser M. (2018). Implementation of nationwide cancer survivorship plans: Experience from the UK. J. Cancer Policy.

[77] Michel G., Mulder R.L., van der Pal H.J.H., Skinner R., Bárdi E., Brown M.C., Vetsch J., Frey E., Windsor R., Kremer L.C.M., Levitt G. (2019). Evidence-based recommendations for the organization of long-term follow-up care for childhood and adolescent cancer survivors: a report from the PanCareSurFup Guidelines Working Group. J. Cancer Survivorsh. Res. Pract..

[78] Strauss S.J., Frezza A.M., Abecassis N., Bajpai J., Bauer S., Biagini R., Bielack S., Blay J.Y., Bolle S., Bonvalot S., Boukovinas I., Bovee J., Boye K., Brennan B., Brodowicz T., Buonadonna A., Álava E., Dei Tos A.P., Garcia Muro X., Dufresne A., Eriksson M., Fagioli F., Fedenko A., Ferraresi V., Ferrari A., Gaspar N., Gasperoni S., Gelderblom H., Gouin F., Grignani G., Gronchi A., Haas R., Hassan A.B., Hecker-Nolting S., Hindi N., Hohenberger P., Joensuu H., Jones R.L., Jungels C., Jutte P., Kager L., Kasper B., Kawai A., Kopeckova K., Krákorová D.A., Cesne A., Grange F., Legius E., Leithner A., López Pousa A., Martin-Broto J., Merimsky O., Messiou C., Miah A.B., Mir O., Montemurro M., Morland B., Morosi C., Palmerini E., Pantaleo M.A., Piana R., Piperno-Neumann S., Reichardt P., Rutkowski P., Safwat A.A., Sangalli C., Sbaraglia M., Scheipl S., Schöffski P., Sleijfer S., Strauss D., Sundby Hall K., Trama A., Unk M., Sande M.A.J., Graaf W.T.A., Houdt W.J., Frebourg T., Ladenstein R., Casali P.G., Stacchiotti S. (2021). Bone sarcomas: esmo-euracan-genturis-ern paedcan clinical practice guideline for diagnosis, treatment and follow-up. Ann. Oncol..

[79] Hale S.C. (2023). British Sarcoma Group Conference, Cardiff, Wales.

[80] James R.P. (2024). British Sarcoma Group Conference, Leeds, UK.

[81] Shuel S.L. (2024). Cardiotoxicity of cancer treatment. Can. Fam. Physician.

[82] Mehta R.L., Kellum J.A., Shah S.V., Molitoris B.A., Ronco C., Warnock D.G., Levin A. (2007). Acute Kidney Injury Network: report of an initiative to improve outcomes in acute kidney injury. Critical Care (London England).

[83] Minejima E., Choi J., Beringer P., Lou M., Tse E., Wong-Beringer A. (2011). Applying new diagnostic criteria for acute kidney injury to facilitate early identification of nephrotoxicity in vancomycin-treated patients. Antimicrob. Agents Chemother..

[84] Guerra E.N.S., Almeida F.T., Bezerra F.V., Figueiredo P., Silva M.A.G., De Luca Canto G., Pachêco-Pereira C., Leite A.F. (2017). Capability of CBCT to identify patients with low bone mineral density: a systematic review. Dentomaxillofac. Radiol..

[85] Noble W. (1991). Assessment of impaired hearing. Pinter Publishers Limited.

[86] Sills E.S., Alper M.M., Walsh A.P. (2009). Ovarian reserve screening in infertility: practical applications and theoretical directions for research. Eur. J. Obstet. Gynecol. Reprod. Biol..

[87] Barratt C.L.R., Björndahl L., De Jonge C.J., Lamb D.J., Osorio Martini F., McLachlan R., Oates R.D., van der Poel S., St John B., Sigman M., Sokol R., Tournaye H. (2017). The diagnosis of male infertility: an analysis of the evidence to support the development of global WHO guidance-challenges and future research opportunities. Hum. Reprod. Update.

[88] Jones J.M., Olson K., Catton P., Catton C.N., Fleshner N.E., Krzyzanowska M.K., McCready D.R., Wong R.K., Jiang H., Howell D. (2016). Cancer-related fatigue and associated disability in post-treatment cancer survivors. J. Cancer Survivorsh. Res. Practice.

[89] McFarlane A.C. (2010). The long-term costs of traumatic stress: intertwined physical and psychological consequences. World Psych. Off. J. World Psych. Assoc. (WPA).

[90] Lewin J., Thompson K., Bae S., Desai J., Strong R., Caruso D., Howell D., Herschtal A., Sullivan M., Orme L. (2017). Variations of surveillance practice for patients with bone sarcoma: A survey of Australian sarcoma clinicians. Sarcoma.

[91] Palmer S., Patterson P., Thompson K. (2014). A national approach to improving adolescent and young adult (AYA) oncology psychosocial care: the development of AYA-specific psychosocial assessment and care tools. Palliat. Support. Care.

